# Branchial cleft cyst: a case report

**DOI:** 10.11604/pamj.2017.26.102.11895

**Published:** 2017-02-28

**Authors:** Moncef Sellami, Abdelmonem Ghorbel

**Affiliations:** 1 Department of Otorhinolaryngology-Head and Neck Surgery Habib Bourguiba, University Hospital, Sfax, Tunisia; 2 Sfax Medical School, University of Sfax, Sfax, Tunisia

**Keywords:** Branchial cleft cyst, neck, surgery

Brachial cleft cysts are rare congenital malformations of the lateral neck and typically present in the second and third decades of life. Many theories have been proposed to explain the aetiology of these cysts including incomplete obliteration of branchial mucosa, persistence of vestiges of the precervical sinus and cystic lymph node origin. Preoperative imaging investigations show a fluid-filled cyst and evaluate anatomic relationships. Differential diagnosis includes metastatic squamous cell carcinoma, lymphadenitis, lymphoma, cervical dermoid cyst, cystic hygroma and parotid pathology. Surgical excision is the treatment of choice and results in a good prognosis. We present the case of a 37-year-old woman referred to our department for assessment of a neck swelling that had gradually increased in size during the previous nine months. The clinical examination found a 6 cm non-tender mass of the upper-left neck. The MRI revealed a cystic lesion measuring 4×5×6 cm, displacing the sternocleidomastoid muscle posterolaterally, and the carotid and internal jugular vein medially. The mass was completely resected through a lateral cervicotomy. The histological examination revealed a cystic wall lined with stratified squamous epithelium consistent with the diagnosis of a branchial cleft cyst. The postoperative course was uneventful. Long-term follow-up has shown no evidence of recurrence.

**Figure 1:**
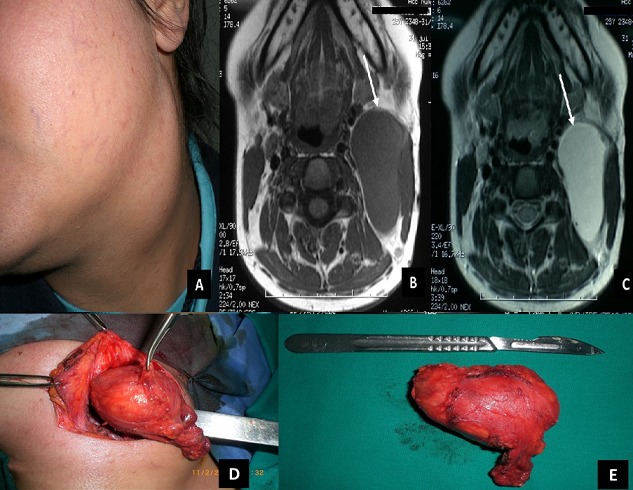
A) clinical image showing a mass of the upper-left neck; B) axial T1-weighted MRI shows a well-defined hypointense mass that was confined to the left aspect of the neck (arrow); C) the mass (arrow) was hyper-intense on T2-weighted sequences (Axial T2-weighted MRI); D) peroperative view; E) the excised mass was an encapsulated cystic structure

